# *MRE11A* Isoform Expression Associated with Outcome Following Radiotherapy in Muscle-Invasive Bladder Cancer does not Alter Cell Survival and DNA Double-Strand Break Repair Following Ionising Radiation

**DOI:** 10.3233/BLC-190209

**Published:** 2019-08-16

**Authors:** Alexandra K. Walker, Juri Na, Lisa Browning, Nada Humayun-Zakaria, Maurice P. Zeegers, K.K. Cheng, Nicholas D. James, Richard T. Bryan, Roland Arnold, Anne E. Kiltie

**Affiliations:** a CRUK/MRC Oxford Institute for Radiation Oncology, University of Oxford, Oxford, UK; b Department of Cellular Pathology, Oxford University Hospitals NHS Foundation Trust, John Radcliffe Hospital, and NIHR Oxford Biomedical Research Centre, Oxford, UK; c NUTRIM School for Nutrition and Translational Research in Metabolism and CAPHRI Care and Public Health Research Institute, Maastricht University, Maastricht, the Netherlands; d The Institute of Cancer and Genomic Sciences, University of Birmingham, Birmingham, UK

**Keywords:** Bladder cancer, *MRE11*, rs1805363, *MRE11* isoform 1, *MRE11* isoform 2

## Abstract

**Background:**

DNA double strand breaks are the cytotoxic lesions produced by ionising radiation. Critical for the repair of these lesions is the DNA damage response protein MRE11 which, in a complex with RAD50 and NBS1, mediates DNA damage signalling and double-strand break repair. We previously found the presence of an *MRE11* germline single nucleotide polymorphism (SNP), rs1805363 (*G* > A), to be associated with poor outcome following radiotherapy (RT) and increased expression of *MRE11* isoform 2 in a limited panel of bladder cancer cell lines and tumours.

**Objectives:**

To look for further evidence in support of the SNP/isoform association in a larger panel of germline and tumour samples donated by patients diagnosed with invasive bladder cancer, and to test the hypothesis that bladder cancer cells expressing *MRE11* isoform 2 would be more radio resistant than cells expressing *MRE11* isoform 1.

**Methods:**

Germline DNA from 189 patients with invasive bladder cancer (141 T2, 48 T1) was genotyped for the rs1805363 *G* > A SNP. Loss of heterozygosity was determined by genotyping tumour DNA in 17GA germline patients. The Cancer Genome Atlas was mined to correlate presence of the GA germline genotype with MRE11 isoform expression. We used colony formation assays and *γ*H2AX foci kinetics after ionising radiation in RT112 MRE11 knockdown cells expressing ectopic *MRE11* isoform 1 or 2.

**Results:**

Of the 189 germline DNA samples, 22 contained both the A minor allele and G major allele with the remaining wild type containing only the G major allele. LOH was identified in seven of 17 available tumour samples. Tumour *MRE11* isoform 2 expression was found to be significantly higher (*p* = 0.007) in patients’s samples containing the A minor allele compared to those with only the G major allele (*n* = 23). In the TCGA database we found 16% (66 out of 406) of bladder tumours heterozygous for the SNP and only two homozygous, and a significant relative increase of isoform 2 usage (*p* = 0.017). We identified no significant difference in radio sensitivity between bladder cancer cells expressing either *MRE11* isoform.

**Conclusions:**

In this study the *MRE11* isoform 2 was not found to be associated with increased cellular sensitivity to radiation. We conclude that the previously reported association between the germline rs1805363 SNP and poor survival in MIBC patients following RT is unlikely to be related to the DNA damage response function of *MRE11* isoform 2.

## INTRODUCTION

Organ-confined muscle-invasive bladder cancer (MIBC) is routinely treated using either radical cystectomy or bladder-sparing approaches including chemoradiotherapy (CRT) or radiotherapy alone, with or without neoadjuvant chemotherapy. Cystectomy and radiotherapy have been shown to result in similar cause-specific survival rates [1] but no randomised trial has successfully compared the two modalities. Predictive biomarkers that could aid treatment decision making would be useful in this setting and could potentially increase patient survival rates.

DNA double strand breaks (DSB) are the cytotoxic lesions caused by ionising radiation and are repaired via two major pathways: non-homologous end joining and homologous recombination [2–4]. MRE11 is a DNA damage response protein which can bind DNA and which possesses endo- and exonuclease activity. Many of the functions of MRE11 are reliant on its ability to form a complex with Nijmegen breakage syndrome 1 (NBS1) and RAD50, the MRN complex. The MRN complex is involved in both the recognition of DSBs, enhancing the rate of ATM activation and subsequent DNA damage signalling [5–9], and the repair of DSBs through its role as a nuclease in homologous recombination [10, 11]. The alternative splicing of *MRE11* results in two transcript variants encoding *MRE11* isoform 1 and *MRE11* isoform 2, respectively. The two *MRE11* isoforms are similar, with the only difference in the translated protein being the absence of a short 27 amino-acid-long exon, exon 16, in *MRE11* isoform 2. At the transcript level *MRE11* isoform 2 has a longer 5’ UTR than the *MRE11* isoform 1.

We have previously shown that high tumour protein levels of MRE11, as determined by immunohistochemistry, were associated with improved patient outcome following radio therapy in MIBC patients [12, 13]. Using next generation sequencing, we also identified a single nucleotide polymorphism (SNP) within *MRE11* (rs1805363) which was associated with worse cancer-specific survival following radiotherapy [14]. The rs1805363 SNP is a germline *MRE11* variant (G to A transition) which is located intronically in *MRE11* isoform 1, five bases 3’ to the exon 1 AG donor splice site, and in *MRE11* isoform 2 is located within the 5’UTR. In a panel of bladder cancer cell lines and three patient tumour samples, the presence of the rs1805363 A minor allele was found to correlate with an increase in the expression of *MRE11* isoform 2 over *MRE11* isoform 1 [14].

Here, we sought to validated our previous finding that the presence of the rs1805363 A minor allele correlates with higher *MRE11* isoform 2 expression in patients with invasive bladder cancer by examining a larger number of tumour samples for the proportion of isoform 2 relative to isoform 1 expression. In addition, we mined variation and expression data from the TCGA bladder cancer cohort for the relationship of *MRE11* isoform usage and rs1805363. We also tested the hypothesis that bladder cancer cells expressing *MRE11* isoform 2 would be more radioresistant than cells expressing *MRE11* isoform 1. We generated cell lines which expressed an *MRE11* construct containing the coding sequence of *MRE11* isoform 1 or *MRE11* isoform 2, and then used the cells in assays to determine cellular radiosensitivity and DSB repair efficiency.

## MATERIALS AND METHODS

### Patient materials

Germline DNA, extracted tumour DNA and FFPE bladder cancer biopsies were obtained from participants in the Bladder Cancer Prognosis Programme (University of Birmingham, UK) under ethical approval (06/MRE04/65) [15] BCPP participants were recruited between 2005 and 2011, and gave informed consent for enrolment into BCPP on the basis of initial cystoscopic findings suggestive of primary bladder cancer. All patients were newly-diagnosed, had not received treatment prior to biospecimen collection, and were subsequently treated and followed-up according to contemporary guidelines (including re-resection where indicated). Inclusion and exclusion criteria are detailed elsewhere [15].

### Genotyping the RS1805363 SNP

A predesigned TaqMan SNP Genotyping assay (C_11474841_10) (Applied Biosystems-Thermo Fisher Scientific, Foster City, CA, USA) was used with a 7500 Fast Real Time PCR system under standard genotyping parameters to genotype patient DNA. Analysis was conducted using 7500 software.

### RNA extraction

Ten-micron thick tissue sections plus an additional 4*μ*m thick tissue section were cut from FFPE bladder cancer biopsies. Four micron sections were H&E stained and sent to a Consultant Uropathologist (LB) to demarcate invasive areas of tumour tissue. Ten micron sections were placed at –20°C and stored until microdissection, with storage at –20°C limited to a maximum of 3 weeks. For each patient sample, invasive tumour tissue was scraped from 10×10*μ*m tissue sections and placed in a sterile Eppendorf tube using a 15C carbon steel sterile scalpel blade (Swann Morton). Macrodissected tissue was then immediately subjected to RNA extraction using an RNeasy FFPE kit (Qiagen, Venlo, Netherlands) with deparaffinisation solution (Qiagen, Venlo, Netherlands). Successful RNA extraction was confirmed by running 5*μ*l of RNA on a 1% agarose (Sigma Aldrich, St. Louis, MO, USA) gel. RNA was converted into cDNA using a High-Capacity cDNA reverse transcription kit with RNase inhibitor (Applied Biosystems-Thermo Fisher Scientific, Waltham, CA, USA).

### Quantification of MRE11 isoforms

Primers were designed (Forward- GGACTTGAAGCATCTACGTT, Reverse- ATGCGATTCCTAAATTACCC) to flank the MRE11 rs1805363 *G* > A SNP, resulting in polymerase chain reaction (PCR) fragment sizes of 144bp for *MRE11* isoform 1 and 251 bp for *MRE11* isoform 2. PCRs were conducted on cDNA using OneTaq Quik-load master mix with standard buffer (New England BioLabs, MA, USA) and products were separated on a 1% agarose (Sigma Aldrich, St. Louis, MO, USA) gel. The gel was visualised using a Molecular Imager Gel Doc XR system (BioRad, Hercules, CA, USA) and the band intensities of the PCR products analysed using Image Lab software (BioRad, Hercules, CA, USA). Primers for GAPDH (Forward- ACAGTCAGCCGCATCTTCTT, Reverse- AATGAAGGGGTCATTGATG) were used in a concurrent PCR and run next to the MRE11 isoform amplification PCR as a control for cDNA input and quality.

### TCGA

We downloaded all RNA sequencing reads mapping to MRE11A from TCGA [16] using the bam-slicing functionality of the GDC data portal. Since the position of the SNP rs1805363 is not covered in the exome capture sequencing available in TCGA, we downloaded the Genome-Wide Human SNP Array 6.0 data which contains rs1805363. High confidence calls for the genotype (Birdseed [17] confidence score <0.1) were extracted (instances not matching the criteria were assigned the label ‘unknown’). In total, we could obtain genotype data for 410 samples with paired RNA sequencing data (all primary cancer, no normal controls or metastatic samples). Isoform usage of the MRE11A locus was then quantified using Salmon (version 0.12.0 using standard parameters [18]) using the MRE11A isoforms described in Ensembl (version 96 [19] and Gencode (version 29), resulting in Transcript per Million Read values (TPM) for each isoform. Of the ten isoforms described, we found five regularly expressed in the TCGA samples. We tested a shift in relative isoform usage of the variant samples (genotype G vs. A) versus wild-type using an unpaired one-sided Wilcoxon test on the relative use of each isoform, and on the pairwise ratios of each isoform against each other.

### Cell culture and transfection

293T cells were obtained from ATCC, Manassas, VA, USA, in 2011 and confirmed to be mycoplasma negative by PCR in October 2016. RT112 cell lines were obtained from DSMZ, Braunschweig, Germany, in 2016. RT112 wildtype, RT112-MRE11 KD, RT112-full length MRE11 and RT112-MRE11 *Δ*exon16 cells (see below) were confirmed to be mycoplasma negative by PCR in September 2018. Cell lines were used up to 20 passages from original stock.

293T cells were maintained in high glucose DMEM (Gibco-Thermo Fisher Scientific, CA, USA) supplemented with 10% FBS (Thermo Fisher, Waltham, CA, USA) without antibiotics. Cells were maintained at 37°C and 5% CO_2_ in a humidified incubator. Cells were transfected in 10 cm dishes seeded at 5x10^6^ cells per dish, and 9*μ*g of either pLenti-full length MRE11 or pLenti-MRE11-*Δ*exon16, 4.5*μ*g of psPAX2 (#12260; Addgene, MA, USA), and 4.5*μ*g of pMD2.G (#12259; Addgene, MA, USA) were delivered to each dish with Lipofectamine 3000 (Thermo fisher, MA, USA) in opti-MEM (Thermo Fisher, Waltham, CA, USA), according to manufacturer’s instructions. The generated lentiviral particles were collected 48 hr following transfection and filtered through a 0.45*μ*m syringe filter (SLHV033RS; Millipore, Burlington, MA, USA). RT112-MRE11 KD cells were generated as previously described [20], and additionally, a monoclonal cell line was isolated from a polyclonal pool of stable cells and expanded. RT112-MRE11 KD cells were infected with the filtered lentiviral supernatant using 8*μ*g/ml of polybrene. After 3 days of infection, 5*μ*g of puromycin was added for selection with complete media (RPMI medium supplemented with 10% FBS). MRE11-mutated stable cell lines were established after 96-well monoclonal selection and samples were collected or fixed at 10 min, 30 min, 2 hr, 6 hr and 24 hr after 2Gy of Cs-137 ionising radiation using a GSR-D1 caesium-137 irradiator (Gamma Services, Surrey, UK).

### Vectors and site-directed mutagenesis

MRE11 sequences were inserted into the vector pLenti-puro-CMV (P100022; Vigene, Rockville, MD, USA). A QuickChange II XL Site-Directed Mutagenesis Kit (#200521; Agilent, Santa Clara, CA, USA) and XL10-Gold ultracompetent cells were used for all site-directed mutageneses according to the manufacturer’s instructions. DNA sequencing (Source Bioscience, Nottingham, UK) was used to confirm the mutated nucleotides and the deleted exon 16 sequences (Supplementary Figure 1).

### Western blotting

Protein lysis buffer was prepared in 50 mmol/L HEPES, 100 mmol/L NaCl, 10 mmol/L EDTA, 1% Triton X-100, 4 mmol/L Na pyrophosphate, 2 mmol/L sodium orthovanadate, 10 nmol/L NaF, and 50 mmol/L B-glycerophosphate. Cells were lysed in lysis buffer containing a cocktail of proteinase inhibitors (Roche, Mannheim, Germany). Protein quantification of the lysates was performed using a BCA protein assay (Thermo Fisher, Waltham, MA, USA) and 30*μ*g of protein was resolved on 4% to 20% polyacrylamide gels and transferred onto nitrocellulose membranes. The resulting membranes were incubated with blocking buffer (Li-cor Biosciences, Lincoln, NE, USA) and primary antibodies. The antibodies used were mouse polyclonal anti-MRE11 (ab214; abcam, Cambridge, UK), rabbit anti-phospho-histone H2A.X (Ser139) (#2577; Cell Signalling Technology, Danvers, MA, USA), and mouse monoclonal anti-*β*-actin (ab6276; abcam, Cambridge, UK). Fluorochrome-conjugated secondary antibodies (Li-cor Biosciences, Lincoln, NE, USA) were used and detected by infrared scanning densitometry using the Li-cor Odyssey Infrared Detection System (Li-cor Biosciences, Lincoln, NE, USA).

### Clonogenic assay

RT112 cells and stable cell lines were plated in 6 cm culture dishes containing 4 ml of fresh medium with appropriate numbers in triplicate (300–1,800) and were irradiated at 0–10 Gy on the day after cell plating and incubated for 2 weeks. Cells were stained with crystal violet staining solution (0.5%) in 80 ml distilled water, 20 ml methanol, and 0.5 g crystal violet powder (Merck, Whitehouse station, NJ, USA). Colonies containing more than 50 cells were counted and the surviving fraction was determined as the total number of colonies formed divided by the total number of cells plated multiplied by the plating efficiency, as determined in untreated cells. Radiation survival curves were plotted in GraphPad Prism, using the linear-quadratic model with the equation SF = exp – (*αD* +*βD^2^*), where D is a dose of radiation.

### Immunofluorescence

RT112 and stable cell lines were plated onto 8-well chamber slides (734–2050; NUNC, Roskilde, Denmark) and fixed with 100% methanol for 5 min at –20°C, at each time point after IR. Then cells were washed three times for 5 min each time in PBS. All subsequent steps were carried out at room temperature. Samples were blocked for 30 min in blocking buffer (1% BSA, 22.52 mg/ml glycine in PBST (PBS + 0.1% Tween 20)). Rabbit anti-phospho-histone H2A.X (Ser139) (#2577; Cell Signalling Technology, Danvers, MA, USA) was diluted 1:500 in PBST and incubated overnight at 4°C. Cells were washed with PBS, after which secondary antibody [AlexaFluor 568 (A11036; Thermo Fisher Scientific, Waltham, MA, USA)] was diluted 1:2000 in PBST and applied for 1 hr in room temperature. Cells were washed with PBS as before and mounted by using proLong diamond antifade mountant with DAPI (P36962; Thermo Fisher Scientific, Waltham, MA, USA). Cells were visualised for *γ*-H2AX immunostaining through confocal microscopy (Zeiss 710).

### Data analysis

Clonogenic assays, western blots, and immunofluorescence data are representative of three independent experiments. For *γ*-H2AX foci kinetics, *P* values were calculated using Kruskal Wallis test with Dunn’s multiple comparisons in GraphPad Prism. Counting of *γ*-H2AX foci was determined using Image J software. For clonogenic assays, surviving fraction was calculated on the basis of the number of colonies on non-irradiated plates. Data are presented as the log of the surviving fraction with error bars representing the SEM.

## RESULTS

We obtained 189 samples of germline DNA from the University of Birmingham’s Bladder Cancer Prognosis Programme which were genotyped for the rs1805363 *G* > A SNP. Of these patients, 141 were diagnosed with muscularis propria invasion (stage T2+) and 48 with lamina propria invasion (T1). Of the 189 samples, 22 were identified as containing both A and G alleles, with the remainder identified as only containing the major G (WT) allele. Loss of heterozygosity (LOH) within heterozygous tumours was investigated by genotyping extracted tumour DNA, which was available for 17 of the germline genotyped casesidentified as positive for both A and G alleles. LOH was identified in 7 of the 17 samples (A only in 4/17, G only in 3/17, A and G in the remaining 10/17).

**Fig.1:**
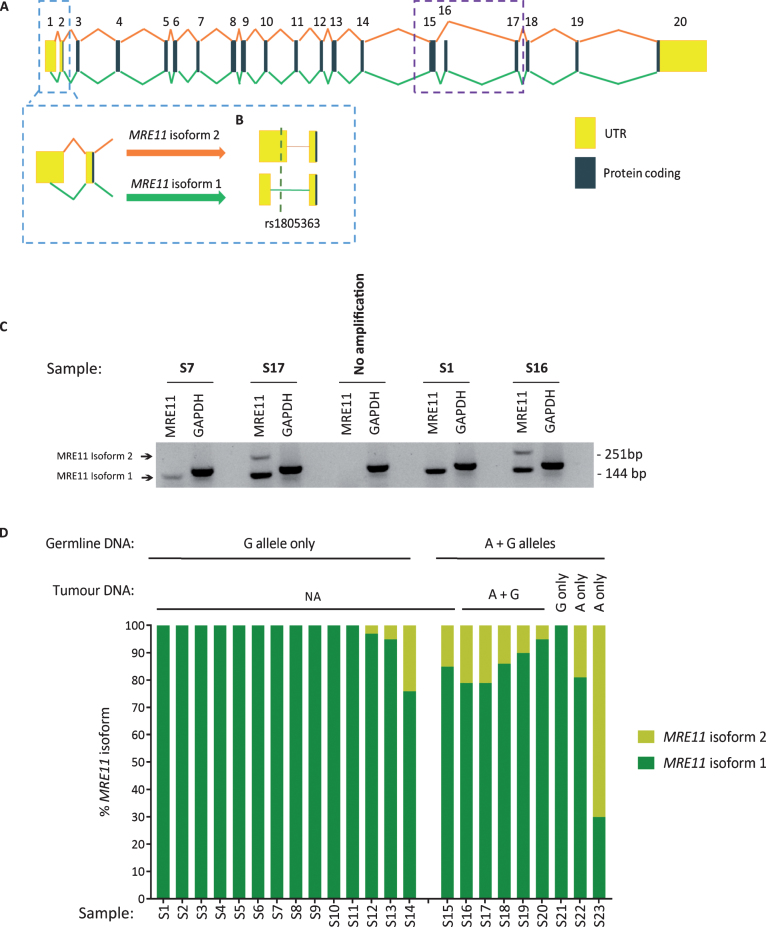
Overview of MRE11 isoform 1 and 2 structure, and the distribution of MRE11 isoformsin tumour samples.(A)Schematic illustrating the splicing of *MRE11* pre-mRNA to form *MRE11* isoform 1 and 2.Exons are displayed as coloured numbered boxes. Yellow and dark blue boxes portray exons within untranslated regions (UTR) and protein coding regions respectively. Green lines directly beneath the exon boxes depict *MRE11*isoform 1 splicing and *MRE11* isoform 2 splicing is described by the orange lines above. The skipping of exon 16 in *MRE11* isoform 2 is highlighted by the purple dotted box. The light blue dotted box highlights the usage of an alternative splice site within exon 1, producing a shorter exon 1 sequence in *MRE11* isoform 1. (B) The relative position of the rs1805363 SNP within the 5’ UTR of both *MRE11* isoforms. Relative exon sizes are based on MRE11A RefSeq accessions NM_005591.3 and NM_005590.3. Relative intron lengths were calculated using BLAT to aligning reference mRNA sequences onto genomic DNA [29]. (C)Representative image of electrophoresis bands produced from the PCRs conducted on cDNA from tumour samples. The 144 bp product is amplified from the *MRE11* isoform 1 5’UTR and the 251 bp product is amplified from the longer *MRE11* isoform 2 5’UTR. Amplification of *GAPDH* was run as a positive control for all samples. (D) Quantification of tumour *MRE11* isoform expression. *MRE11* isoform 1 and 2 expression is displayed as a percentage of the total amount of *MRE11* in each sample. Samples are grouped according to the presence or absence of the rs1805363 A minor allele in germline DNA. The tumour genotype is displayed where known. The proportion of *MRE11* isoform 2 expression is significantly increased in patients who are positive for the rs1805363 A minor allele compared to patients who are only positive for the G major allele (*t*-test *p* = 0.007).

**Fig.2:**
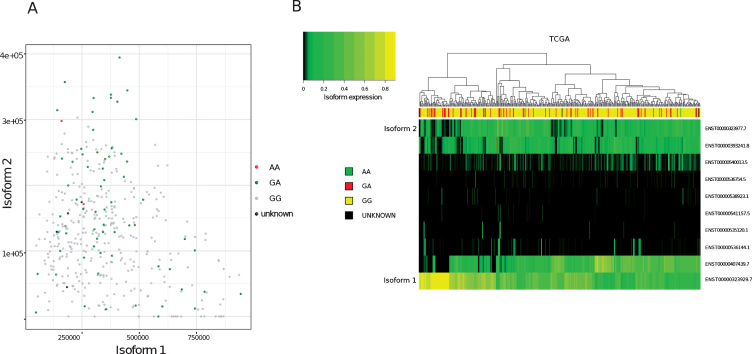
Analysis of MRE11 isoforms in TCGA bladder cancer tumours (A)Expression values (Transcript per million, TPM, computed on reads mapping to MRE11 only) of isoform 1 (x-axis) compared to isoform 2 (y-axis) in TCGA bladder cancer samples. Node colours indicate variant calling for rs1805363.(B)Clustering of MRE11 isoforms. Colour intensity refers to percentage of isoform usage.

To determine the relative proportions of isoform 1 and isoform 2 in patients’ tumours, FFPE tumour blocks were obtained for RNA extraction from 36 of the germline genotyped samples. Twelve of the tumour blocks were not suitable for RNA extraction. This was due to either a lack of invasive pathology in cut sections (6/12), the area of tissue being too small for sufficient material to be collected (5/12) or the tissue being identified as highly inflamed (1/12). Of the remaining 24 blocks, 10 were from patients’ germline positive for both A and G alleles and 14 from patients positive for only the G (WT) allele. RNA extracted from patient tumours was then used to quantify the relative expression of isoform 1 and 2 within the samples and grouped according to germline genotype (Fig. 1). One sample was excluded from the analysis due to a failure in isoform amplification. In agreement with previous findings, *MRE11* isoform 2 expression was found to be significantly higher (*p* = 0.007) in patients positive for the rs1805363 A minor allele (mean proportion of *MRE11* isoform 2 in the germline genotyped patient group containing both A and G alleles: 19.4%) compared to patients positive for only the G major allele (mean proportion of *MRE11* isoform 2 in the G only germline genotyped patient group: 2.3%). Isoform 2 was also found to be generally increased in non-wild-type samples in TCGA (*p*-value: <0.001), as well as in direct comparison to isoform 1 (*p* = 0.017) (see Supplementary Table 1). Although there was a trend for variant SNPs to correlate with isoform 2 expression, not all samples followed this trend (Fig. 2A). Furthermore, a clustering based on the expression of all isoforms did not separate wild-type from variant samples (Fig. 2B).

We then investigated whether the increased presence of *MRE11* isoform 2 could influence cellular radiosensitivity and DNA damage response. We used an RT112 bladder cancer cell line knocked down for endogenous MRE11 and re-expressing an MRE11 construct containing either the full length MRE11 coding sequence (*MRE11* isoform 1) or the MRE11 coding sequence lacking MRE11 exon 16 (*MRE11* isoform 2). As this question referred to the function of the translated protein we did not include the *MRE11* isoform 1 or 2 5’UTR sequences in these constructs.

**Fig.3:**
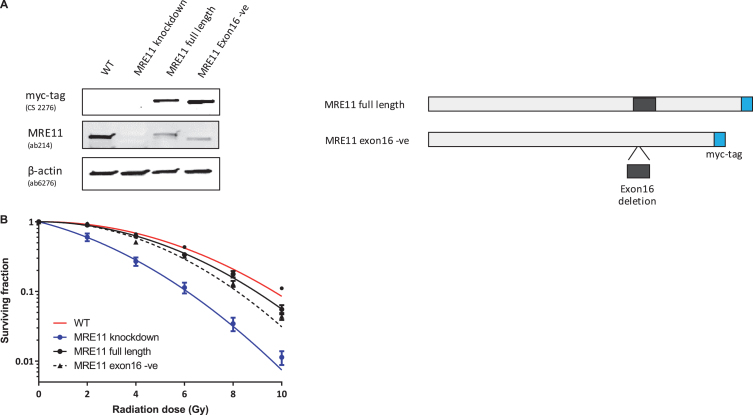
Establishment of MRE11 knockdown, MRE11 full length, and MRE11 exon16 -ve cell lines, and their clonogenic survival after irradiation(A) Western blot of RT112 cells and RT112-MRE11 knockdown cells stably transduced with lentiviruses containing either MRE11 full length or MRE11 exon16 –ve sequences and myc tag. (B) Clonogenic survival assays were performed following ionising radiation and surviving curves were fitted using the linear-quadratic equation.

Lenti-viral expression of either *MRE11* isoform 1 or isoform 2 in these cells led to a greater than 2.5-fold increase in full-length MRE11 protein level (2.61-fold for MRE11 isoform 1, 2.53-fold for MRE11 isoform 2), which corresponds to approximately 30% of the endogenous full-length MRE11 level in the parental cell line (Fig. 3A).

**Fig.4:**
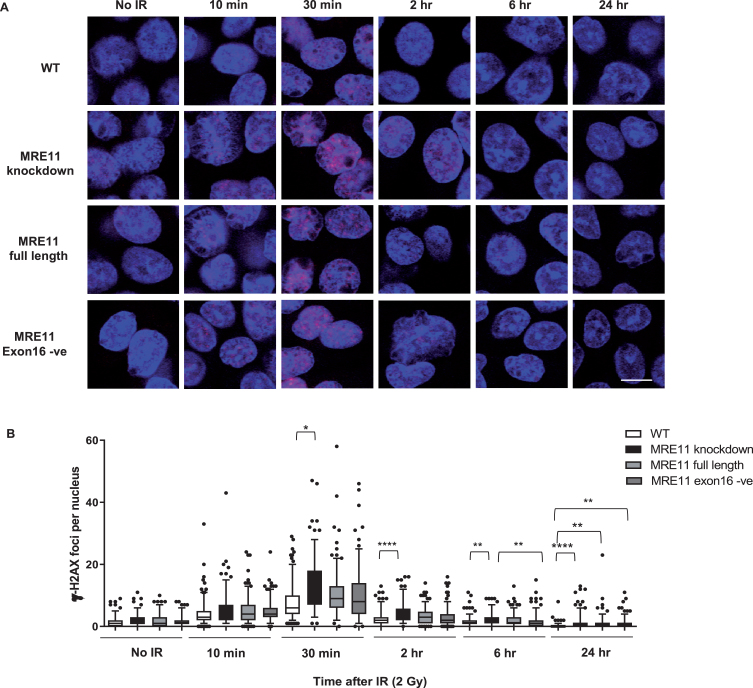
Radiation-induced formation of *γ*-H2AX foci in MRE11 full length and MRE11 exon16 -ve rescued cells(A) RT112 cells were treated with 2 Gy irradiation and incubated at 37°C for the times shown. Representative images are displayed with*γ*-H2AX foci in redand blue represents DAPI staining (scale bar: 10*μ*m). (B) Statistical analyses of number of *γ*-H2AX foci per cell are shown as box and whisker plots with 5th–95th percentiles. In total, 180 nuclei were counted per sample in 3 independent experiments. Asterisks indicate ^*^P≤0.05, ^**^ *P*≤0.01, ^***^P≤0.001, and ^****^P≤0.0001.

A colony survival assay was performed to determine the radiosensitivity of the MRE11 RT112 cell lines. The MRE11 knockdown cell line displayed a significant increase in radiosensitivity compared to the parental RT112 cell line at doses of 2 to 10 Gy. This was rescued by the re-expression of either the MRE11 full length (isoform 1) (1.6-fold and 1.9-fold reduction of parental cell survival levels at 8 and 10 Gy) or the MRE11 exon16-negative construct (isoform 2) (1.6-fold and 2.3-fold reduction of parental cells at 8 and 10 Gy), while MRE11 knockdown cells had 62.5-fold and 52.6-fold reduction on survival compared to parental cells at 8 and 10 Gy. However, there was no significant difference in radiosensitivity between cells expressing the full-length version MRE11 and exon16-deleted (*Δ*exon16) cells (Fig. 3B). To determine whether *Δ*exon16 cells had an altered functional response to DSB,*γ*-H2AX foci were quantified after exposure to 2Gy of IR. The level of *γ*-H2AX started to increase 10 min after 2Gy IR regardless of MRE11 expression. It reached its peak at 30 minutes post-IR, with significantly (1.81-fold) higher levels in the MRE11 knockdown cells compared to the parental cell line that persisted for 2, 6 and 24 hrs after irradiation. Consistent with the level of expression achieved compared to the parental cells, MRE11-transfected cells showed a partial reduction in *γ*-H2AX foci at 2 hr after 2 Gy irradiation, at an intermediate level between the parental and MRE11 knockdown cells. The reduction in *γ*-H2AX after rescue with either the MRE11 full length or *Δ*exon16 constructs was 1.3-fold at 30 minutes after 2 Gy IR, similar to the MRE11 protein levels achieved relative to the parental cells. There was no significant difference in *γ*-H2AX foci at any of the time points between the MRE11 full length and the *Δ*exon16 cells (Fig. 4A and B). Moreover, the initial *γ*-H2AX foci level (No IR) for all four cell lines were re-achieved at the 6 hr time point. This suggests that the absence of exon 16 has minimal effects on cell survival and repair of DNA damage after IR.

## DISCUSSION

In line with our previous report on the *MRE11* rs1805363 SNP in patients with invasive bladder cancer, we genotyped the rs1805363 status of germline DNA and in this study provided further evidence for a correlation between the presence of the *MRE11* germline rs1805363 A minor allele and an increase in the tumour expression of *MRE11* isoform 2 over isoform 1. However, it would be useful to establish if the tumour genotype is a more accurate indicator of the expression of *MRE11* isoform 2 in invasive bladder tumours.

In TCGA, the SNP variant was even more frequent (at 16% minor allele frequency) than reported earlier (e.g. 11% in Teo et al; lower frequencies in different variation studies as reported in dbGAP, including approximately 5% in the TopMed meta-study and approximately 8% in the European population (see Supplementary Table 1). However, it is not possible to determine whether patients with cancer have a higher frequency of the SNP in their germline or whether this result reflects the somatic nature of the TCGA data. The isoform quantification of the TCGA data clearly supports the observation of a variant-dependent increase of isoform 2, but not all samples follow this trend. The bioinformatics approach to quantify the isoforms relied on the full set of annotated isoforms (in total 10) which, while allowing a more complete picture, might also hamper the analysis, since the algorithm might not be able to distinguish all isoforms correctly in all instances. The results of the TCGA analysis therefore highlights the fact that further research is required to determine how to best quantify the *MRE11* isoforms from RNA-seq data. In addition, it would be of interest to study other isoforms, as for example ENST00000540013.5 coding for a much shorter protein without exon 16, which seems to be supressed in the non-wild-type cases (Supplementary Table 1).

Given that the presence of the rs1805363 A minor allele in the germline DNA of patients with invasive bladder cancer was associated with worse survival after radiotherapy [14], we hypothesised that cells expressing increased levels of *MRE11* isoform 2 may have an altered response to ionising radiation compared to cells expressing *MRE11* isoform 1. However, when we re-expressed constructs for *MRE11* isoform 1 or 2 in a bladder a cancer cell line which had been knocked down for endogenous MRE11 and used assays to assess their intrinsic radiosensitivity, no difference was observed between cells expressing the full length coding sequence for MRE11 (*MRE11* isoform 1) or cells expressing MRE11 lacking exon 16 (*MRE11* isoform 2). We therefore conclude that the presence or absence of *MRE11* exon 16 may not influence cell survival or DSB repair capacity after IR, contrary to our initial hypothesis, although we appreciate that our conclusions are limited by the use of only one cell line system.

In our cellular analysis of *MRE11* isoform 2 we were interested in directly comparing the protein function of our MRE11 constructs. Therefore, we selected cells for our assays that stably re-expressed our constructs. Unfortunately, this strategy did not allow us to address whether there was a difference in the protein stability or turnover between the two isoforms. Ideally, the protein levels of *MRE11* isoform 1 and isoform 2 would have been assessed in patient samples where we had quantified the relative expression of mRNA. However, unfortunately with no available antibody distinguishing between the two isoforms and with only a 27aa difference between the two protein variants, it was not possible to reliably distinguish the isoforms using techniques such as western blotting or immunohistochemistry. We did have an antibody raised to exon 16 but this unfortunately proved unsuitable for western blotting, and so we could not verify its specificity for use in determining the isoforms’ presence in the samples. Interestingly, the phosphorylation of MRE11 threonine 597, which resides within exon16, has been found to promote MRE11 degradation in PTEN deficient cells [21], which does suggest that in some cases a difference in protein stability may exist between the two isoforms.

A limitation in the system we used to investigate the efficiency of DSB repair by *MRE11* isoform 1 compared to 2 is that we only re-expressed either *MRE11* isoform 1 or 2 in our KD cells. However, in the human tissue we analysed *MRE11* isoform 1 was present in conjunction with isoform 2. Therefore this method may be an oversimplification of the underlying biology and expressing different ratios of the two isoforms may be a more representative model in which to study their function. Indeed, in cells, MRE11 exists as a homodimer with dimerisation being critical for the correct assembly of the MRN complex and the binding and aligning of DNA ends [22]. It is possible that although each isoform in this analysis was proficient for DSB repair when expressed in isolation, a dimer comprised of the two different isoforms may not be. The complexity of the interactions between MRE11 and the members of the MRN complex have been previously highlighted. It has been proposed that as the MRN complex is a heterohexamer comprised of 3 protein dimers it could exist in multiple states, where different dimer assemblages within the complex affect its response to DNA damage [23].

Aside from its role in DNA damage repair, MRE11 also functions in the repair of stalled and collapsed replication forks [24–28]. While we saw no defect in the ability of MRE11 lacking exon16 to repair DNA damage following ionising radiation, it may be that this form of MRE11 is not able to function in response to replication stress. This would be worthy of further investigation and a possible reason for the poor response of rs1805363 positive patients with high *MRE11* isoform 2 levels to radiotherapy.

## FUNDING

The authors report no funding.

## CONFLICT OF INTERESTS

The authors have no conflict of interest to report.

## AUTHOR CONTRIBUTIONS

AW performed experiments, interpreted the data and drafted and revised the manuscript

JN performed experiments, interpreted the data and drafted and revised the manuscript

LB provided histopathological expertise for tumour identification and interpretation, and revised the manuscript

RA performed bioinformatics analysis, interpreted the data and drafted and revised the manuscript

N H-Z performed bioinformatics analysis and interpreted the data

MZ contributed patient samples

KC contributed patient samples

NJ contributed patient samples

RB contributed patient samples, interpreted the data and revised the manuscript

AK conceived the study, supervised the work and revised the manuscript.

All authors approved the final version of the manuscript and agreed to be accountable for the accuracy and integrity of the work.

## Supplementary Material

Supplementary Material

## References

[1] Kotwal S , Choudhury A , Johnston C , Paul AB , Whelan P , Kiltie AE . Similar treatment outcomes for radical cystectomy and radical radiotherapy in invasive bladder cancer treated at a United Kingdom specialist treatment center. Int J Radiat Oncol Biol Phys. 2008;70(2):456–63. 17904301 10.1016/j.ijrobp.2007.06.030

[2] Shrivastav M , De Haro LP , Nickoloff JA . Regulation of DNA double-strand break repair pathway choice. Cell Res. 2008;18(1):134–47. 18157161 10.1038/cr.2007.111

[3] Ceccaldi R , Rondinelli B , D’Andrea AD . Repair Pathway Choices and Consequences at the Double-Strand Break. Trends Cell Biol. 2016;26(1):52–64. 26437586 10.1016/j.tcb.2015.07.009 PMC4862604

[4] Haber JE . Partners and pathwaysrepairing a double-strand break. Trends Genet. 2000;16(6):259–64. 10827453 10.1016/s0168-9525(00)02022-9

[5] Carson CT , Schwartz RA , Stracker TH , Lilley CE , Lee DV , Weitzman MD . The Mre11 complex is required for ATM activation and the G2/M checkpoint. EMBO J. 2003;22(24):6610–20. 14657032 10.1093/emboj/cdg630 PMC291825

[6] Lee JH , Mand MR , Deshpande RA , Kinoshita E , Yang SH , Wyman C , et al Ataxia telangiectasia-mutated (ATM) kinase activity is regulated by ATP-driven conformational changes in the Mre11/Rad50/Nbs1 (MRN) complex. J Biol Chem. 2013;288(18):12840–51. 23525106 10.1074/jbc.M113.460378 PMC3642328

[7] Dupre A , Boyer-Chatenet L , Gautier J . Two-step activation of ATM by DNA and the Mre11-Rad50-Nbs1 complex. Nat Struct Mol Biol. 2006;13(5):451–7. 16622404 10.1038/nsmb1090

[8] Lee JH , Paull TT . Direct activation of the ATM protein kinase by the Mre11/Rad50/Nbs1 complex. Science. 2004;304(5667):93–6. 15064416 10.1126/science.1091496

[9] Lee JH , Paull TT . ATM activation by DNA double-strand breaks through the Mre11-Rad50-Nbs1 complex (vol 308, pg 551, 2005). Science. 2005;308(5730):1870. 15790808 10.1126/science.1108297

[10] Anand R , Ranjha L , Cannavo E , Cejka P . Phosphorylated CtIP Functions as a Co-factor of the MRE11-RAD50-NBS1 Endonuclease in DNA End Resection. Mol Cell. 2016;64(5):940–50. 27889449 10.1016/j.molcel.2016.10.017

[11] Shibata A , Moiani D , Arvai AS , Perry J , Harding SM , Genois MM , et al DNA double-strand break repair pathway choice is directed by distinct MRE11 nuclease activities. Mol Cell. 2014;53(1):7–18. 24316220 10.1016/j.molcel.2013.11.003 PMC3909494

[12] Laurberg JR , Brems-Eskildsen AS , Nordentoft I , Fristrup N , Schepeler T , Ulhoi BP , et al Expression of TIP60 (tat-interactive protein) and MRE11 (meiotic recombination 11 homolog) predict treatment-specific outcome of localised invasive bladder cancer. BJU Int. 2012;110(11 Pt C):E1228–36. 23046361 10.1111/j.1464-410X.2012.11564.x

[13] Choudhury A , Nelson LD , Teo MT , Chilka S , Bhattarai S , Johnston CF , et al MRE11 expression is predictive of cause-specific survival following radical radiotherapy for muscle-invasive bladder cancer. Cancer Res. 2010;70(18):7017–26. 20843819 10.1158/0008-5472.CAN-10-1202 PMC2941719

[14] Teo MT , Dyrskjot L , Nsengimana J , Buchwald C , Snowden H , Morgan J , et al Next-generation sequencing identifies germline MRE11A variants as markers of radiotherapy outcomes in muscle-invasive bladder cancer. Ann Oncol. 2014;25(4):877–83. 24623370 10.1093/annonc/mdu014 PMC3969555

[15] Zeegers MP , Bryan RT , Langford C , Billingham L , Murray P , Deshmukh NS , et al The West Midlands Bladder Cancer Prognosis Programme: Rationale and design. BJU Int. 2010;105(6):784–8. 19751260 10.1111/j.1464-410X.2009.08849.x

[16] Cancer Genome Atlas Research N, Weinstein JN , Collisson EA , Mills GB , Shaw KR , Ozenberger BA , et al The Cancer Genome Atlas Pan-Cancer analysis project. Nat Genet. 2013;45(10):1113–20. 24071849 10.1038/ng.2764 PMC3919969

[17] Korn JM , Kuruvilla FG , McCarroll SA , Wysoker A , Nemesh J , Cawley S , et al Integrated genotype calling and association analysis of SNPs, common copy number polymorphisms and rare CNVs. Nat Genet. 2008;40(10):1253–60. 18776909 10.1038/ng.237 PMC2756534

[18] Patro R , Duggal G , Love MI , Irizarry RA , Kingsford C . Salmon provides fast and bias-aware quantification of transcript expression. Nat Methods. 2017;14(4):417–9. 28263959 10.1038/nmeth.4197 PMC5600148

[19] Zerbino DR , Achuthan P , Akanni W , Amode MR , Barrell D , Bhai J , et al Ensembl 2018. Nucleic Acids Res. 2018;46(D1):D754–D61. 29155950 10.1093/nar/gkx1098 PMC5753206

[20] Nicholson J , Jevons SJ , Groselj B , Ellermann S , Konietzny R , Kerr M , et al E3 Ligase cIAP2 Mediates Downregulation of MRE11 and Radiosensitization in Response to HDAC Inhibition in Bladder Cancer. Cancer Res. 2017;77(11):3027–39. 28363998 10.1158/0008-5472.CAN-16-3232

[21] Piscitello D , Varshney D , Lilla S , Vizioli MG , Reid C , Gorbunova V , et al AKT overactivation can suppress DNA repair via p70S6 kinase-dependent downregulation of MRE11. Oncogene. 2018;37(4):427–38. 28967905 10.1038/onc.2017.340 PMC5799716

[22] Williams RS , Moncalian G , Williams JS , Yamada Y , Limbo O , Shin DS , et al Mre11 dimers coordinate DNA end bridging and nuclease processing in double-strand-break repair. Cell. 2008;135(1):97–109. 18854158 10.1016/j.cell.2008.08.017 PMC2681233

[23] Williams GJ , Lees-Miller SP , Tainer JA . Mre11-Rad50-Nbs1 conformations and the control of sensing, signaling, and effector responses at DNA double-strand breaks. DNA Repair (Amst). 2010;9(12):1299–306. 21035407 10.1016/j.dnarep.2010.10.001 PMC3008338

[24] Hashimoto Y , Ray Chaudhuri A , Lopes M , Costanzo V . Rad51 protects nascent DNA from Mre11-dependent degradation and promotes continuous DNA synthesis. Nat Struct Mol Biol. 2010;17(11):1305–11. 20935632 10.1038/nsmb.1927 PMC4306207

[25] Kolinjivadi AM , Sannino V , De Antoni A , Zadorozhny K , Kilkenny M , Techer H , et al Smarcal1-Mediated Fork Reversal Triggers Mre11-Dependent Degradation of Nascent DNA in the Absence of Brca2 and Stable Rad51 Nucleofilaments. Mol Cell. 2017;67(5):867–81 e7. 28757209 10.1016/j.molcel.2017.07.001 PMC5594205

[26] Schlacher K , Christ N , Siaud N , Egashira A , Wu H , Jasin M . Double-strand break repair-independent role for BRCA2 in blocking stalled replication fork degradation by MRE11. Cell. 2011;145(4):529–42. 21565612 10.1016/j.cell.2011.03.041 PMC3261725

[27] Yang Y , Liu Z , Wang F , Temviriyanukul P , Ma X , Tu Y , et al FANCD2 and REV1 cooperate in the protection of nascent DNA strands in response to replication stress. Nucleic Acids Res. 2015;43(17):8325–39. 26187992 10.1093/nar/gkv737 PMC4787816

[28] Ying S , Hamdy FC , Helleday T . Mre11-dependent degradation of stalled DNA replication forks is prevented by BRCA2 and PARP1. Cancer Res. 2012;72(11):2814–21. 22447567 10.1158/0008-5472.CAN-11-3417

[29] Kent WJ . BLAT–the BLAST-like alignment tool. Genome Res. 2002;12(4):656–64. 11932250 10.1101/gr.229202 PMC187518

