# Supplementary material for: MRE11A Isoform Expression Associated with Outcome Following Radiotherapy in Muscle-Invasive Bladder Cancer does not Alter Cell Survival and DNA Double-Strand Break Repair Following Ionising Radiation
Source: Bladder Cancer. 2019 Aug 16;5(2):147–57. doi: 10.3233/BLC-190209 (PMC6949534; doi:10.3233/BLC-190209)
Supplement: Supplementary Material [file blc-5-blc190209-s001.docx]

**Supplementary Figure 1. Mre11 exon 16 was deleted using site-directed mutagenesis and confirmed by Sanger DNA sequencing**

Alignment of Sequences: [Translation of MRE11_Full length] with [Translation of MRE11 Δexon16]

Similarity : 679/708 (95.90 %)

MRE11 FL MSTADALDDENTFKILVATDIHLGFMEKDAVRGNDTFVTLDEILRLAQENEVDFILLGGD 60

||||||||||||||||||||||||||||||||||||||||||||||||||||||||||||

MRE11 Δexon16 MSTADALDDENTFKILVATDIHLGFMEKDAVRGNDTFVTLDEILRLAQENEVDFILLGGD 60

MRE11 FL LFHENKPSRKTLHTCLELLRKYCMGDRPVQFEILSDQSVNFGFSKFPWVNYQDGNLNISI 120

||||||||||||||||||||||||||||||||||||||||||||||||||||||||||||

MRE11 Δexon16 LFHENKPSRKTLHTCLELLRKYCMGDRPVQFEILSDQSVNFGFSKFPWVNYQDGNLNISI 120

MRE11 FL PVFSIHGNHDDPTGADALCALDILSCAGFVNHFGRSMSVEKIDISPVLLQKGSTKIALYG 180

||||||||||||||||||||||||||||||||||||||||||||||||||||||||||||

MRE11 Δexon16 PVFSIHGNHDDPTGADALCALDILSCAGFVNHFGRSMSVEKIDISPVLLQKGSTKIALYG 180

MRE11 FL LGSIPDERLYRMFVNKKVTMLRPKEDENSWFNLFVIHQNRSKHGSTNFIPEQFLDDFIDL 240

||||||||||||||||||||||||||||||||||||||||||||||||||||||||||||

MRE11 Δexon16 LGSIPDERLYRMFVNKKVTMLRPKEDENSWFNLFVIHQNRSKHGSTNFIPEQFLDDFIDL 240

MRE11 FL VIWGHEHECKIAPTKNEQQLFYISQPGSSVVTSLSPGEAVKKHVGLLRIKGRKMNMHKIP 300

||||||||||||||||||||||||||||||||||||||||||||||||||||||||||||

MRE11 Δexon16 VIWGHEHECKIAPTKNEQQLFYISQPGSSVVTSLSPGEAVKKHVGLLRIKGRKMNMHKIP 300

MRE11 FL LHTVRQFFMEDIVLANHPDIFNPDNPKVTQAIQSFCLEKIEEMLENAERERLGNSHQPEK 360

||||||||||||||||||||||||||||||||||||||||||||||||||||||||||||

MRE11 Δexon16 LHTVRQFFMEDIVLANHPDIFNPDNPKVTQAIQSFCLEKIEEMLENAERERLGNSHQPEK 360

MRE11 FL PLVRLRVDYSGGFEPFSVLRFSQKFVDRVANPKDIIHFFRHREQKEKTGEEINFGKLITK 420

||||||||||||||||||||||||||||||||||||||||||||||||||||||||||||

MRE11 Δexon16 PLVRLRVDYSGGFEPFSVLRFSQKFVDRVANPKDIIHFFRHREQKEKTGEEINFGKLITK 420

MRE11 FL PSEGTTLRVEDLVKQYFQTAEKNVQLSLLTERGMGEAVQEFVDKEEKDAIEELVKYQLEK 480

||||||||||||||||||||||||||||||||||||||||||||||||||||||||||||

MRE11 Δexon16 PSEGTTLRVEDLVKQYFQTAEKNVQLSLLTERGMGEAVQEFVDKEEKDAIEELVKYQLEK 480

MRE11 FL TQRFLKERHIDALEDKIDEEVRRFRETRQKNTNEEDDEVREAMTRARALRSQSEESASAF 540

||||||||||||||||||||||||||||||||||||||||||||||||||||||||||||

MRE11 Δexon16 TQRFLKERHIDALEDKIDEEVRRFRETRQKNTNEEDDEVREAMTRARALRSQSEESASAF 540

MRE11 FL SADDLMSIDLAEQMANDSDDSISAATNKGRGRGRGRRGGRGQNSASRGGSQRGRADTGLE 600

||||||||||||||||||||||||||||||||||||||||||||||||||||||######

MRE11 Δexon16 SADDLMSIDLAEQMANDSDDSISAATNKGRGRGRGRRGGRGQNSASRGGSQRGR------ 594

MRE11 FL TSTRSRNSKTAVSASRNMSIIDAFKSTRQQPSRNVTTKNYSEVIEVDESDVEEDIFPTTS 660

#######################|||||||||||||||||||||||||||||||||||||

MRE11 Δexon16 -----------------------FKSTRQQPSRNVTTKNYSEVIEVDESDVEEDIFPTTS 631

MRE11 FL KTDQRWSSTSSSKIMSQSQVSKGVDFESSEDDDDDPFMNTSSLRRNRR 708

||||||||||||||||||||||||||||||||||||||||||||||||

MRE11 Δexon16 KTDQRWSSTSSSKIMSQSQVSKGVDFESSEDDDDDPFMNTSSLRRNRR 679

**Supplementary Table 1. Enrichment analysis and additional isoform information used in the TCGA analysis.** Sheet 1: pairwise enrichment analysis (P-values of Wilcoxon on-sided unpaired test on increase of relative isoform abundance of mutant vs. WT, pairwise for each isoform). Sheet 2: Overview on used isoform annotations for quantification and P-values for increased relative abundance of one isoform (P-values of Wilcoxon on-sided unpaired test on increase of relative isoform abundance mutant vs. WT, each isoform against all other isoforms). Sheet 3: overview of allele frequencies in different studies from dbGAP.
